# Knee arthroplasty in Denmark, Norway and Sweden: A pilot study from the Nordic Arthroplasty Register Association

**DOI:** 10.3109/17453671003685442

**Published:** 2010-03-31

**Authors:** Otto Robertsson, Svetlana Bizjajeva, Anne Marie Fenstad, Ove Furnes, Lars Lidgren, Frank Mehnert, Anders Odgaard, Alma Becic Pedersen, Leif Ivar Havelin

**Affiliations:** ^1^The Swedish Knee Arthroplasty Register, Dept of Orthopedics, Clinical Sciences, Lund. Lund UniversitySweden; ^2^Swedish National Musculoskeletal Competence Centre (NKO), LundSweden; ^3^The Norwegian Arthroplasty Register, Department of Orthopaedic Surgery, Haukeland University Hospital, BergenNorway; ^4^Department of Surgical Sciences, Faculty of Medicine and Dentistry, University of BergenNorway; ^5^Danish Knee Arthroplasty Register and Competence Centre North, Department of Clinical Epidemiology, Aarhus University HospitalDenmark; ^6^The Danish Knee Arthroplasty Register and Department of Orthopaedics, Aarhus University HospitalDenmark

## Abstract

**Background and purpose:**

The number of national arthroplasty registries is increasing. However, the methods of registration, classification, and analysis often differ.

**Methods:**

We combined data from 3 Nordic knee arthroplasty registers, comparing demographics, methods, and overall results. Primary arthroplasties during the period 1997–2007 were included. Each register produced a dataset of predefined variables, after which the data were combined and descriptive and survival statistics produced.

**Results:**

The incidence of knee arthroplasty increased in all 3 countries, but most in Denmark. Norway had the lowest number of procedures per hospital—less than half that of Sweden and Denmark. The preference for implant brands varied and only 3 total brands and 1 unicompartmental brand were common in all 3 countries. Use of patellar button for total knee arthroplasty was popular in Denmark (76%) but not in Norway (11%) or Sweden (14%). Uncemented or hybrid fixation of components was also more frequent in Denmark (22%) than in Norway (14%) and Sweden (2%).

After total knee arthroplasty for osteoarthritis, the cumulative revision rate (CRR) was lowest in Sweden, with Denmark and Norway having a relative risk (RR) of 1.4 (95% CI: 1.3–1.6) and 1.6 (CI: 1.4–1.7) times higher. The result was similar when only including brands used in more than 200 cases in all 3 countries (AGC, Duracon, and NexGen). After unicompartmental arthroplasty for osteoarthritis, the CRR for all models was also lowest in Sweden, with Denmark and Norway having RRs of 1.7 (CI: 1.4–2.0) and 1.5 (CI: 1.3–1.8), respectively. When only the Oxford implant was analyzed, however, the CRRs were similar and the RRs were 1.2 (CI: 0.9–1.7) and 1.3 (CI: 1.0–1.7).

**Interpretation:**

We found considerable differences between the 3 countries, with Sweden having a lower revision rate than Denmark and Norway. Further classification and standardization work is needed to permit more elaborate studies.

## Introduction

The Swedish Knee Arthroplasty Register (SKAR) started in 1975 as the first national arthroplasty register in the world, and was followed by the Swedish Hip Arthroplasty Register in 1979. The Finnish Arthroplasty Register was initiated in 1980 and the Norwegian Arthroplasty Register (NAR) in 1987, although it started with registration of knees in 1994. The Danish Hip Arthroplasty Register was established in 1995 and the Danish Knee Arthroplasty Register (DKR) in 1997. Since the start of these pioneering registries, several other national registries have followed around the world. On an international level, there has been an effort to increase cooperation of the existing registries in order to standardize methods and introduce common definitions of terms used in registry settings, as well as to encourage registry work where none existed before.

Because the Nordic registers were the early starters and the respective countries have similar health organizations, personal identity numbers and census registers, they should obviously be the ones that are most easily combined and compared. In order to accomplish this, the NARA (Nordic Arthroplasty Register Association) was started in 2007. This cooperation has resulted in an attempt to produce and analyze a combined dataset from the hip and knee arthroplasty databases in Denmark (DK), Norway (NO), and Sweden (SE). The initial findings of the hip project have recently been published (Havelin et al. 2009). For administrative reasons, Finland was not part of this initial project.

There are potential benefits to combining data from different countries in order to reveal possible differences with respect to disease patterns, methods, and results. In addition, being able to analyze a larger material is helpful from a statistical point of view when one is interested in uncommon disorders or methods. As the methods of registration and classification are different, however, there are some hurdles to be overcome before datasets can be combined and analyzed.

We investigated whether a common dataset on knee arthroplasties could be produced, that would permit initial comparisons of incidence, population characteristics, and outcomes.

## Methods

For this pilot study, a NARA working group was set up in order to agree on a dataset containing defined variables that all the registers could provide. For implant and cement brands, however, each register provided its own national definitions to be classified at a later stage.

The register from each country produced its own dataset containing the variables agreed upon, while de-identifying patients by replacing their personal ID number with a unique serial number. Hospital names were similarly de-identified. The data were sent to Lund, Sweden where the datasets were combined and where the different classifications of brand of implant were combined into common implant definitions.

Knee arthroplasties entered during the period 1997–2007 were included. The start of the period coincides with the start of the DKR. Incidence numbers from SE and NO were available from the start of the respective national registers in 1975 and 1994.

### Statistics

When calculating the median number of surgeries per hospital and year, each year was evaluated separately so that the number varied depending on what year was being evaluated. In cases where a hospital had not performed any surgeries during a year, that year did not count.

Incidence of knee arthroplasty was calculated for each country using population information available on the websites of the national census registers (www.dst.dk, www.ssb.no, and www.scb.se) and the registered number of arthroplasties. Furthermore, an age-standardized incidence was calculated for a standard “European” population (Waterhouse et al. 1976).

Revision performed for any reason was the endpoint in the survival analyses, with revision being defined as exchange, removal, or addition of prosthetic components.

Cumulative revision rate (CRR) curves were produced using the life table method with monthly intervals. The confidence intervals (CIs) were calculated using the Wilson quadratic equation with Greenwood and Peto effective sample-size estimates (Dorey et al. 1993). Curves were cut off when 40 knees remained at risk.

When comparing risk of revision between countries, Cox regression was used and relative risk (RR) estimates with 95% confidence interval (CI) were calculated. Adjustment was made for differences in sex, age category (< 45, 45–54, 55–64, 65–74, 75–84, and ≥ 85), and year of operation. When only the 3 most commonly used TKA models were analyzed, adjustment was also made for use of implant model.

Significance level was set to 95%. Statistical analyses were carried out using the PASW statistics package version 18 (SPSS Inc., Chicago, IL).

## Results

During the study period (1997–2007), a total of 151,814 primary knee arthroplasties were inserted in the 3 countries (Table 1). The surgeries were performed at 52 hospitals in DK, 82 in NO, and 99 in SE. The median number of surgeries performed at each hospital per year was highest in DK and lowest in NO (Table 2).

**Table 1.** Characteristics of knee arthroplasty patients and operative methods as registered in the NARA database, for the period 1997–2007

	Denmark	Norway	Sweden
No. of knee arthroplasties	38,411	26,451	86,952
No. of hospitals	52	82	99
% females	64	68	62
% OA	82	85	92
% RA	4.3	5.9	4.7
% Other	14	9	3.4
TKA	35,569	23.096	76.304
% females	64	70	63
Mean age at TKA	69	70	71
Femur & tibia cemented	78	86	98
Femur & tibia uncemented	7.7	1.8	1.2
Femur & tibia hybrid	14	12	0.4
% TKA with patellar button	76	11	14
UKA	2,481	3,297	10,157
% females	58	59	57
Mean age at UKA	63	65	66
Femur & tibia cemented	99	99	100
Femur & tibia uncemented	1.20	0	
Femur & tibia hybrid	0.2	0.5	0
Medial UKA	99	48	98
Lateral UKA	0.5	1.4	2.2
Laterality unknown	0	51	0
Fem-Pat	197	52	150
% females	69	71	77
Mean age at femoro-patellar	60	52	64
Other or unknown type	164	6	341

**Table 2.** Numbers of arthroplasties per hospital and year during the period 1997–2007

	Denmark	Norway	Sweden
TKA			
Mean	146	70	143
Median	132	53	105
Range	1–345	1–275	1–527
UKA			
Mean	35	19	30
Median	29	16	24
Range	1–85	1–57	1–104

**Figure 1.**
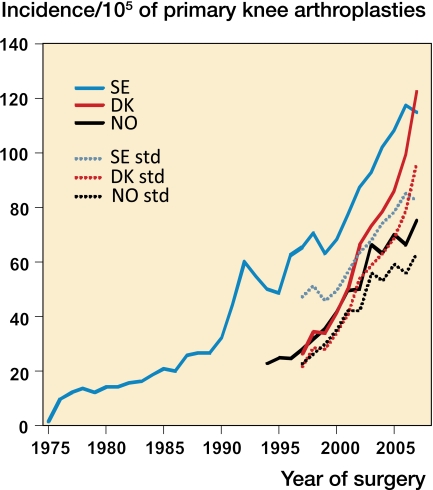
Incidence of primary knee arthroplasties. The solid lines show the incidence and the dotted lines show the age-standardized incidence for the “European” standard population (Waterhouse et al. 1976). Note that due to reduced coverage, the Danish incidence was approximately10–15% higher than shown here.

There was a continuous increase in the overall incidence of registered primary knee arthroplasties per 10^5^ inhabitants in all 3 countries (Table 3). However, in the last 5 years there was a considerably higher increase in DK than in NO and SE (Figure 1). As the age distribution in the 3 countries was fairly similar, age standardization for a standard “European” population (Waterhouse et al. 1976) had little effect on the relative changes in incidence between countries (Figure 1).

**Table 3.** Age- and sex-specific incidence rates of primary total knee arthroplasty per 100,000 inhabitants for the period 1997–2007. Note that due to reduced coverage, the Danish numbers have been underestimated by approximately 10–15%

	Denmark		Norway		Sweden	
Age	Women	Men	Women	Men	Women	Men
< 45	3	2	2	1	2	1
45–54	47	29	32	22	44	27
55–64	169	119	145	85	193	137
65–74	328	227	332	170	450	326
75–84	374	232	349	186	460	323
≥ 85	113	100	79	54	96	91

**Figure 2.**
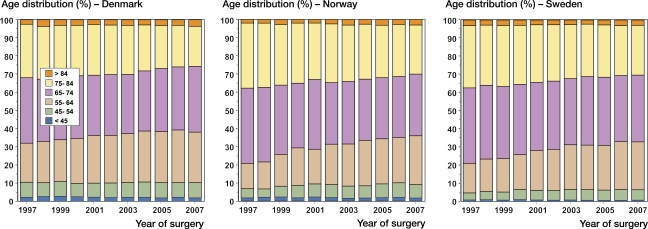
Proportion of age groups each year for primary knee arthroplasty.

In all 3 countries, there was an increase in the proportion of younger patients (between 55 and 64 years of age), particularly in DK—which had the highest proportion of patients less than 65 years (Figure 2).

64% of the operations were on females, with NO having the highest proportion.

The proportion of women was lowest for UKA (58%), higher for TKA (64%), and highest for isolated patello-femoral arthroplasty (PFA) (72%). The mean age at surgery for TKA was 70 years, for UKA it was 66 years, and for PFA it was 60 years. For TKA and UKA, the mean age at surgery was lowest in DK but it was lowest for PFA in NO (Table 1).

**Figure 3.**
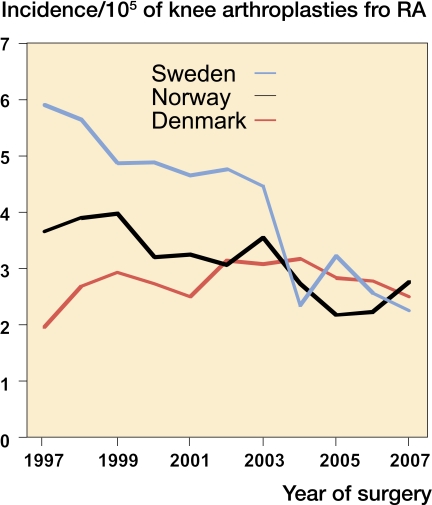
Incidence of arthroplasty for rheumatoid arthritis. Note that due to reduced coverage, the Danish incidence was probably 10–15% higher than shown here.

Osteoarthritis (OA) was the cause of surgery in 88% of all cases, and rheumatoid arthritis (RA) in 5% of all cases. The relative proportion of surgeries for RA decreased during the period in all 3 countries, due to the increase in the number of OA patients. However, the real number of surgeries—and therefore the incidence of RA—also decreased sharply in SE and somewhat in NO, but stayed unchanged in DK (Figure 3).

**Figure 4.**
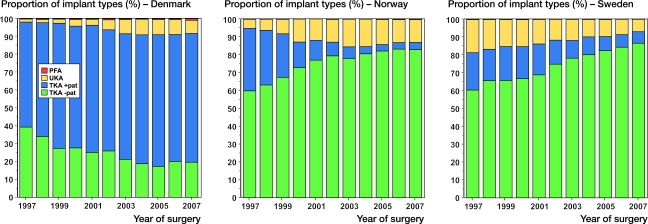
Proportion of implant types used for primary knee arthroplasty.

TKA was the most popular procedure, and was used in 89% of cases. UKA was used for 11% of cases, least often in DK and most often in NO. PFA implants were uncommon, accounting for 0.3% of all procedures. They were used most frequently in DK where they constituted 0.5% of all procedures (Figure 4 and Table 1).

Use of the patellar button in TKA was common in DK, where it was used in 76% of cases. In contrast, the button was only used in 11% and 14% of the TKAs in NO and SE, respectively.

Looking at the trend over the whole time period regarding the use of UKA and TKA with or without patellar button, there were different tendencies in the different countries. Overall, the proportion of UKA increased in DK and NO while it decreased in SE (Figure 4). Use of the patellar button in TKA increased in DK but it decreased in NO and SE.

**Figure 5.**
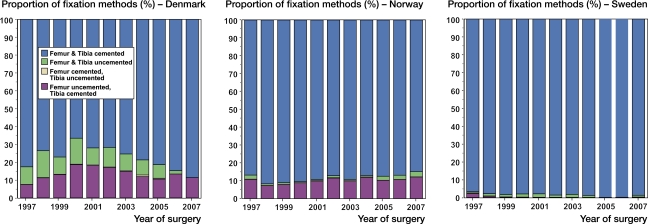
Proportion of fixation methods for primary TKA.

In TKA, cement was used to fix both the tibial and femoral components in 78–98% of cases, least often in DK and most often in SE. Uncemented fixation of both the tibial and the femoral components was most common in DK (7.7%) and much less so in NO (1.8%) and SE (1.2%). Hybrid tibia and femur (mainly with femur uncemented) was more common in DK (14%) and NO (12%) than in SE (0.4%) (Figure 5). In UKA, cemented fixation was the rule in all countries with both the tibial and the femoral components being cemented in 99–100% of cases.

Regarding the choice of TKA implant brands, it appears that DK and SE had somewhat similar preferences while NO was different with 3 of its 5 most popular TKAs being used infrequently in the 2 other countries. Only 3 TKA models (AGC, Duracon, and NexGen) were used in more than 200 cases in each of the 3 countries (Table 4). For UKA, the Oxford implant accounted for 87% and 77% of the surgeries in DK and NO, respectively, while it accounted for 13% in SE. This UKA implant was the only one where more than 100 were used in each of the 3 countries (Table 5).

**Table 4.** The ten most commonly used TKA models in each country during the period 1997–2007

Denmark		Norway		Sweden	
PFC	12,644	PROFIX	7,490	PFC	21,793
AGC	9,774	LCS	7,189	AGC	17,647
NexGen	4,101	AGC	3,129	NexGen	11,809
Maxim	1,466	Genesis	2,530	Duracon	8,455
Advance	1,436	NexGen	972	Free-Sam	8,103
AMK	1,022	Duracon	626	Kinemax	2,205
Vanguard	672	E-motion	402	Scan	1,518
Duracon	593	Kinemax	168	Profix	950
Genesis	131	Tricon	199	Triathlon	673
Kinemax	120	Interax	106	AMK	540

**Table 5.** The five most commonly used UKA models in each country during the period 1997–2007

Denmark		Norway		Sweden	
Oxford	1,683	Oxford	2,566	Link-Uni	4,308
Miller Galante	66	Miller Galante	281	Miller Galante	2,664
PFC Uni	60	Genesis	225	Oxford	1,356
Preservation	48	Preservation	126	Genesis	586
Repicci II	34	MOD III	54	PFC	352

**Figure 6.**
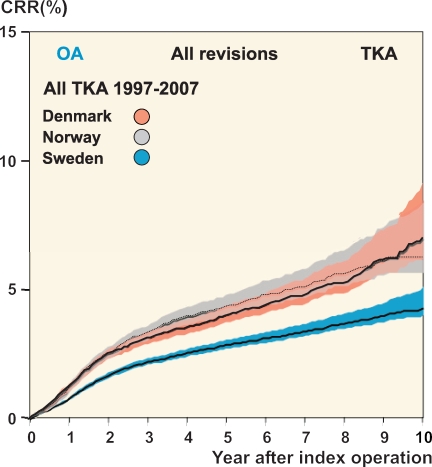
Cumulative revision rate (CRR; lines) with 95% CI (colored areas) after total knee arthroplasty performed for osteoarthritis.

The overall cumulative revision rate (CRR) for TKA and UKA, with any revision as endpoint, was compared in the 3 countries. Including all TKA models inserted for OA, the overall CRR was higher in DK and NO than in SE (Figure 6). Using Cox regression to adjust for differences in age, sex, and year of surgery, the risk ratio (RR) for DK and NO in the case of all TKAs was 1.4 (CI: 1.3–1.6) and 1.6 (CI: 1.4–1.7) times that for Sweden.

**Figure 7.**
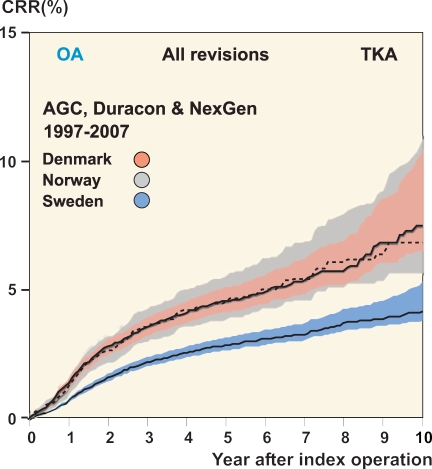
Cumulative revision rate (CRR; lines) with 95% CI (colored areas) for osteoarthritis cases only including the 3 implant brands that were used frequently in all 3 countries.

This was also true when only the 3 models commonly used in all 3 countries (AGC, Duracon, and NexGen) were analyzed (Figure 7). Examining only OA and adjusting for age, sex, year of surgery, and also the implant brand used, DK and NO still had increased RRs compared to SE (RR = 1.6, CI: 1.4–1.8 for DK; RR = 1.7, CI: 1.4–2.0 for NO).

**Figure 8.**
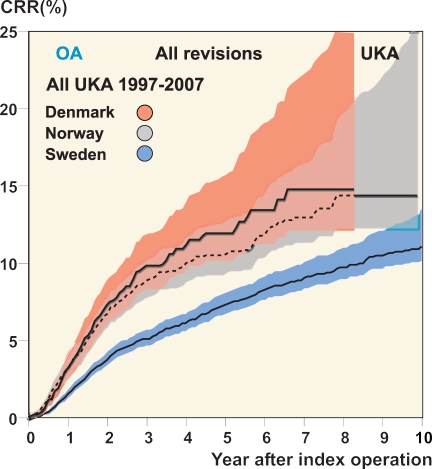
Cumulative revision rate (CRR; lines) with 95% CI (colored areas) after unicompartmental knee arthroplasty performed for osteoarthritis.

**Figure 9.**
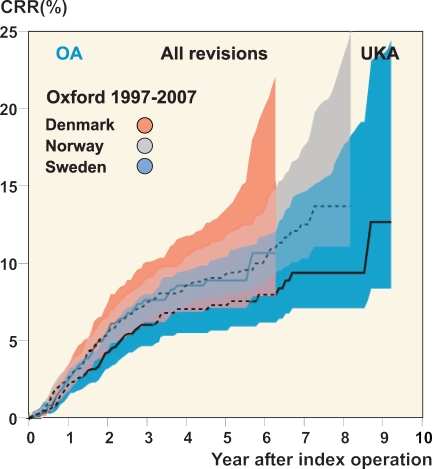
Cumulative revision rate (CRR; lines) with 95% CI (colored areas) for the Oxford unicompartmental implant inserted for osteoarthritis.

In the case of UKA for OA, the CRR for all models was also higher in DK and NO than in SE (Figure 8). The Cox regression also showed similar results, with DK and NO having a risk ratio of 1.7 (CI: 1.4–2.0) and 1.5 (CI: 1.3–1.8), respectively. However, when only the Oxford implant was examined, the CRR was similar at 1.2 (CI: 0.9–1.7) and 1.3 (CI: 1.0–1.7), respectively (Figure 9).

## Discussion

We found that in spite of the similar populations and healthcare systems, there were substantial dissimilarities between the 3 Nordic countries.

When considering the results, it is important to know whether the number of operations in the registers reflects the true number of operations performed. In NO, the number of operations in the NAR was recently checked against the national patient register (NPR)—an in-patient database maintained by the authorities (Espehaug et al. 2006). The NAR was found to contain 99% and 97% as many primary and revision cases, respectively, as registered in the NPR. In SE, a validation and update of the SKAR was performed in 1997, after which the estimated coverage with respect to revisions was estimated to be 94% (Robertsson et al. 1999). A check against the Swedish NPR in 2007 found that the SKAR had more surgeries than the NPR. However, as there were patients in the NPR who had not been registered in SKAR and vice versa, the coverage of SKAR was estimated to be 96% (SKAR annual report 2009). In DK, a similar check has been performed by the DKR, which is the newest register of the 3. The coverage in 2007 was estimated to be 89% for primary surgeries and 81% for revisions (DKR annual report 2009). Thus, there are differences with respect to coverage that may have some effect when the countries are compared. The registers are all prospective, however, and as long as there is no bias in reporting, the effect of reduced coverage only affects precision—but not validity. Although unbiased reduction in coverage of a register reduces the number of cases available for analysis, it does not affect the findings otherwise. Reduced coverage increases the risk of bias occurring, however, as the missing operations may not be randomly distributed between hospitals.

Knee arthroplasty as a routine procedure started earlier in SE than in DK and NO. In 1997, at the start of the observation period, the age-standardized incidence of primary knee arthroplasty in DK and NO was approximately half of that in SE. However, between 1997 and 2007 the standardized incidence increased 4.4 times in DK, compared to 2.8 in NO and 1.8 in SE. Thus, in 2007 the incidence based on reported cases had become higher in DK (123/10^5^) than in both NO (75/10^5^) and SE (115/10^5^). Considering the differences in coverage mentioned above, the incidences for DK should be upgraded by approximately 10–15% for comparison, or to 140/10^5^ in 2007 (DKR annual report 2009).

In the USA, there were approximately 450,000 primary knee replacements in 2005 (Kurtz et al. 2007) and in Australia there were 32,500 in 2007 (AOANJRR annual report 2008). With their populations of 290 million and 22 million, respectively, at the time, this was an incidence of approximately 155/10^5^ and 148/10^5^ with even further increase being projected in the USA (Kurtz et al. 2007). Thus, it seems that future increases can be expected in the Nordic countries, especially in Norway.

The median number of arthroplasties performed by the hospitals each year varied. DK had the highest number of operations per hospital and year. In DK, half of the TKA and UKA procedures were performed at hospitals that, for the year of surgery, inserted more than 132 TKAs and 29 UKAs, respectively. NO had the lowest number of operations per hospital, with half of the TKAs and UKAs being inserted at hospitals performing less than 53 TKAs and 16 UKAs, respectively, per year. Considering that it has been shown for UKA that less than 23 procedures per hospital and year is associated with inferior outcome (Robertsson et al. 2001), this may be less than optimal.

During the study period, the proportion of younger patients increased slightly in all 3 countries. This pattern has been seen elsewhere in the world, such as in the USA (Memtsoudis et al. 2009) and Canada (CIHI 2006). It may be that an increased prevalence of obesity has increased the need for arthroplasty in younger patients and/or that increased confidence in the longevity and results of knee arthroplasty has convinced surgeons that it is safe to use it in younger patients. In addition, findings indicating that—at least in hip arthroplasty—delaying surgery may have a negative effect on outcome (Espehaug et al. 1998, Hajat et al. 2002) may have resulted in surgeons deciding to proceed with surgery in patients with less pain and better function than before. Since several studies have found that younger patients have an increased failure rate (Santaguida et al. 2008), this tendency may increase the need for revisions in future.

Knee arthroplasty was more common in females than in males. In NO, the proportion of females was higher than in DK and SE, which is similar to what has been found for hip arthroplasties (Lohmander et al. 2006, Havelin et al. 2009). It has been speculated that the higher proportion of hip arthroplasty in Norwegian women is due to an increased frequency of childhood hip diseases. However, this is an unlikely explanation regarding knee arthroplasty.

In all 3 countries, surgeries for OA were the reason for the increased volumes.

For RA, the number of surgeries during the period was relatively unchanged in DK while there was a slight reduction in NO and a substantial reduction in SE. A reduction in orthopedic surgery for RA has been reported previously in SE and NO (Weiss et al. 2006, Fevang et al 2007), but it is unclear why the reduction is much greater in SE. There are several possible reasons: a change in disease pattern, a change in prevalence, differences in treatment with immunosuppressive and/or biologic drugs, or that the longer history of knee arthroplasty in SE has emptied the waiting lists of RA patients.

Most of the operations were performed using a TKA. However, while the proportion of UKAs increased slightly in DK and NO over the period, it decreased in SE. This must be seen in the light of the fact that historically, UKA has been popular in SE—which started the period using UKA for almost 20% of knee replacements 1997. On the other hand, DK and NO increased their use of UKA after starting from a very low level.

The reason for UKA being so popular in SE is probably that knee arthroplasty surgery started there in the early 1970s, before modern TKA became widely available. Thus, surgeons had become well-acquainted with UKA by the time that the modern TKA became an alternative.

The use of the patellar button, as well as the fixation methods for primary TKA, also varied between the 3 countries. A patellar button was commonly used in DK (in 76% of TKAs), but only infrequently in NO and SE (11% and 14%). The use of a button became more frequent over time in DK, while the opposite was true of NO and SE.

In all 3 countries, cementing of both the femoral and the tibial components was the most popular method of fixation, and was used for 98% of the cases in SE. However, in DK uncemented or hybrid fixation was used in 22% of cases, and in 14% of cases in NO. With respect to the use of patellar button and uncemented fixation, it appears that DK adheres more to the mid-European and North American customs than the 2 other countries. However, it is not only in the Nordic countries that the choice of methods varies. The Australian Arthroplasty Register (AOANJRR annual report 2008) has reported differences between states and territories regarding the use of both patellar button and fixation methods.

There were considerable differences between the 3 countries in the selection of implant brands. This was also found in a previous NARA study on hips (Havelin et al. 2009).

The question is to what extent the choices are affected by scientific evidence, experience, educational environment, and tradition—and how much they are affected by marketing policies governed by the manufacturers. Whatever the cause, 3 popular TKAs used in NO were hardly used in DK and SE and the predominant UKA brand in NO and DK ranked third in Sweden.

This pilot study was also performed to investigate the degree to which a minimal dataset, including similar information from all 3 registers, could be produced. It turned out that the common classification was probably too restrictive, which, in combination with the decision not to include information on the type of revision surgery, prohibited any in-depth analysis. However, we were able to do a gross comparison of the revision rate using revision for any reason as endpoint. This showed that the revision rate after TKA was lower in SE than in DK and NO, both when analyzing all implants and when analyzing only the 3 implants commonly used in all 3 countries. The revision rate for all UKA implants was also lower in SE. However, when only the implants used in all 3 countries were considered, the difference was smaller and was not statistically significant.

There are many possible reasons for the differences in revision rate observed between the countries. Indications for primary replacement and revision may differ between countries, but there are no data available to substantiate this. The differences in coverage mentioned above might have some influence, as well as the use of different methods and implants. However, considering that SE with its low CRR has a good coverage and that for TKA it did not matter whether all implants or only the 3 implants commonly used in all the countries were taken into account, it appears that this is not the only explanation. Due to the limitations of the initial dataset mentioned above, we decided to suspend further detailed analysis in this respect until further coordination of the datasets has taken place.

Whatever the reasons for these differences, it is an interesting coincidence that the CRR for TKA in NO and DK was similar to what was found in SE for an earlier 8-year time period (1990–1999) (SKAR annual report 2001). The fact that knee arthroplasty became common in SE earlier than in DK and NO might indicate that there is a learning curve involved as a new surgical procedure gains momentum.

Our study has shown that there is a need for further work in producing more detailed and standardized information on a number of variables, in order to permit more elaborate studies. However, the present data showed considerable differences between the 3 countries. The incidence of knee arthroplasty has evolved differently, and the mean number of procedures performed at each hospital varied. The choice of brand of implant was quite different in the 3 countries, as was the use of a patellar button and the type of component fixation. The observed variation in revision rate between these 3 similar countries is surprising and warrants further studies.

One of the main purposes of registers is to observe differences and generate hypotheses. We hope that this collaboration will result in further standardization of registration methods and analyses so that we can return with more research results in future. This would improve our understanding of knee replacement surgery and benefit our patients.
